# Complete and early response to cemiplimab associated to severe immune toxicity in advanced cervical cancer: a case report

**DOI:** 10.3389/fimmu.2023.1303893

**Published:** 2023-12-13

**Authors:** Anna Passarelli, Carmela Pisano, Elisabetta Coppola, Jole Ventriglia, Sabrina Chiara Cecere, Marilena Di Napoli, Luciano Carideo, Secondo Lastoria, Sandro Pignata

**Affiliations:** ^1^ Department of Urology and Gynecology, Istituto Nazionale Tumori Istituto di Ricovero e Cura a Carattere Scientifico (IRCCS) Fondazione G. Pascale, Naples, Italy; ^2^ Nuclear Medicine Unit, Istituto Nazionale Tumori Istituto di Ricovero e Cura a Carattere Scientifico (IRCCS) Fondazione G. Pascale, Naples, Italy

**Keywords:** cemiplimab, immunotherapy, cervical cancer, immune-related adverse events, cardiotoxicity, hepatotoxicity, spleen immune activity

## Abstract

Cervical cancer (CC) is the second most commonly diagnosed cancer and the third leading cause of cancer death among females. The options of treatment for recurrent/advanced CC are limited and patients experiencing recurrence after first line platinum-based chemotherapy have a poor prognosis. In this context, immune checkpoint inhibitors (ICI)s antagonizing PD-1 and programmed death-ligand 1 (PD-L1) have profoundly changed the treatment scenario and outcomes in CC in the first or subsequent lines both as monotherapies or in combination with chemotherapy or other ICIs. Herein, we report the clinical case of a 74-year-old woman with metastatic CC with negative tumor PD-L1 expression who having disease progression after first-line of systemic treatment with platinum, thus undergoing to anti-PD-1 namely cemiplimab. The patient achieved a surprising, fast and complete metabolic response to cemiplimab immediately discontinued after only two cycles due to the onset of rare and severe immune-related adverse events (irAE)s such cardiovascular toxicity and hypertransaminasemia. Despite this, thirteen months later, the patient remains disease-free despite cemiplimab was withdrawn.

## Introduction

Globally, cervical cancer (CC) is the second most commonly diagnosed cancer and the third leading cause of cancer death among females (1). The most significant cause of CC is persistent papillomavirus infection (2).

Metastatic or recurrent CC is usually a symptomatic and devastating condition for the patient (3, 4). The combination of paclitaxel plus cisplatin showed the highest response rate (29%), median PFS (5.8 months) and median OS (12.8 months) and was considered the preferred regimen (5). The combination of paclitaxel and carboplatin could be considered a valid alternative (6). The addition of anti-angiogenic drug as bevacizumab to combination chemotherapy in first line showed an improvement of 3.7 months in median overall survival (OS) (7). Despite the improvement in OS conferred by anti-angiogenic therapy, most patients have progression after first-line and have limited treatment options (8).

To this regard, the background for employing immunotherapy in CC is strongly supported be several features such as HPV-driven carcinogenesis, high tumor mutational burden (TMB), T-cell infiltration, and microsatellite instability (MSI). In addition, the integrated genomic and molecular characterization has amply reported the amplifications in PD-L1 and PD-L2 in CC specimens (9).

In October 2021, the Food and Drug Administration (FDA) granted approval to pembrolizumab in combination with platinum-based chemotherapy with or without bevacizumab in patients with persistent, recurrent, or metastatic PD-L1-positive (CPS≥1) CC (10).

Unfortunately, there was no survival benefit with second-line systemic therapy (8). About this, accelerated approval for pembrolizumab monotherapy for the treatment of patients with recurrent or metastatic CC with disease progression on or after chemotherapy whose tumors express PD-L1 (CPS≥1) was granted in June 2018, based on results from the phase II KEYNOTE-158 basket trial (11, 12).

Cemiplimab, another anti-PD-1 monoclonal antibody, showed significant activity results in pre-treated advanced CC population. In the phase III EMPOWER-Cervical 1 trial, cemiplimab led to significantly longer OS and PFS than standard chemotherapy among patients with recurrent CC who had disease progression after first line platinum-chemotherapy regardless of histology and previous bevacizumab exposure (13). Patients were enrolled regardless of PD-L1 expression. Interestingly objective responses were seen also in patients with PD-L1 expression of less than 1%. The good safety profile of cemiplimab was consistent with that previously reported for the other ICIs in other tumor types (14). Importantly, only 8.7% of patients receiving cemiplimab discontinued treatment for any grade of immune-related adverse events (irAE)s.

Therefore, in September 2021, cemiplimab was granted priority review by the FDA for patients with recurrent or metastatic CC who are not candidates for surgery. In November 2022, also the European Medicines Agency (EMA) has approved cemiplimab (Libtayo®) for the treatment of recurrent or metastatic CC that has progressed on or after platinum-based chemotherapy regardless of PD-L1 expression status or tumor histology.

Although comparative data are lacking, immunotherapy with anti-PD-1 such as pembrolizumab, cemiplimab, and nivolumab is generally expected to show similar clinical activity (15).

Herein, we report an advanced CC case pre-treated with platinum-based regimen that responded favorably to cemiplimab after only two cycles. The patient immediately discontinued immunotherapy due to the onset of rare irAE such cardiovascular toxicity.

Notably, in our patient a complete response to cemiplimab was observed notwithstanding the tumor PD-L1 expression of less than 1%.

To our knowledge, this is the first clinical case of a patient with advanced CC successfully treated with cemiplimab obtaining a complete and early metabolic response in association to severe irAEs.

## Case presentation

In February 2022, a 74-year-old woman was diagnosed with G3 adenosquamous cell carcinoma of the uterine cervix, International Federation of Gynecology and Obstetrics (FIGO) clinical stage IIIA.

Radiological staging with computed tomography (CT) scan at baseline showed a large cervical mass of 5 cm associated with intense hyperaccumulation of the 18-fluorodeoxyglucose (FDG) at to positron emission tomography (PET) examination.

Her family history was negative. Prior personal history was complicated by several important comorbidities such as coronary vasculopathy without significant stenosis, chronic cerebral vascular disease in a patient with a previous ischemic stroke, Von Willebrand disease, chronic obstructive pulmonary disease in a patient strong smoker, and dyslipidemia. Despite that, the patient was functioning well, as indicated by an Eastern Cooperative Oncology Group performance status of 1.

The patient received from April to May 2022 a definitive external beam radiotherapy (EBRT) with a dose of 45Gy/25 fractions and subsequently brachytherapy with a dose of 28 Gy/4 fractions completed in June 2022. The patient obtained a complete radiological and metabolic response following radical radiotherapy treatment on the site of cervical tumor.

Approximately one month after completion of radiotherapy, the patient showed a clinical disease progression with the appearance of a subcutaneous nodule in the right knee that was radically removed. The histological examination confirmed the diagnosis of CC metastases.

The staging with 18-FDG PET scan showed disease progression with several bilateral lung metastases and also subcutaneous nodulation of the left foot.

Following multi-disciplinary discussion and cardiovascular assessment including coronary angiography without evidence of relevant stenosis, the patient was treated with carboplatin (AUC 5) as first-line systemic therapy for a total of three cycles from July to September 2022. Paclitaxel and bevacizumab therapy were excluded for multiple comorbidities.

**Figure 1:**
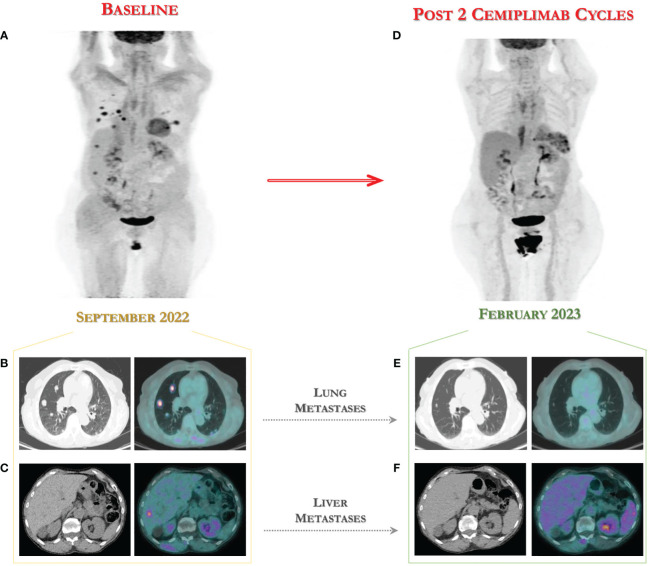
18-FDG PET/CT for assessing the fast tumor response to immunotherapy with cemiplimab. **(A)** Baseline 18-FDG PET images in advanced CC patient showing multiple lung **(B)**, liver **(C)** and lymph node metastases. **(D)** Early assessment after two cycles of cemiplimab showed a metabolic complete response to immunotherapy with disappearing of lung metastases **(E)** and of two hepatic nodules **(F)** to 18-FDG PET scan performed in February 2023.

In September 2022, a new metabolic assessment revealed disease progression with multiple lung metastases to the anterior segment of the right upper lobe (12x11 mm and 18x17 mm) with maximum standardized uptake value (SUVmax) of 9.1 and 7.7 respectively, to the anterior segment of the left lower lobe (17x14 mm) with SUVmax 7.8, new appearance of hyperaccumulation in two hypodense hepatic lesions in the VII and VI segments (SUVmax 5.7), and a lymphadenopathy to the right hilar region (SUVmax 5.3) (see **Figure 1**).The combined positive score (CPS) for PD-L1 on tumor tissue from the last surgery was negative. Given the unavailability of clinical trials in our Institute for this patient, following approval by the Institute’s ethics committee, immunotherapy with cemiplimab, a PD-1-blocking antibody, was provided for compassionate use in a managed access program.

In October 2022, the patient started treatment with cemiplimab at the standard dose of 350 mg every 3 weeks until disease progression or unacceptable toxicity. The immunotherapy was performed for a total of two cycles and then withdrawn due to the onset of severe toxicity.

During the course of cemiplimab treatment, after the first cycle, the patient experienced asymptomatic toxicity of grade 1 such as hepatic adverse event in the form of elevation of alanine aminotransferase (ALT) and aspartate transaminase (AST), for which continued immunotherapy with close laboratory monitoring.

Unfortunately, 14 days after the second cemiplimab infusion, the patient was urgently hospitalized for acute anterior ST-elevation myocardial infarction (STEMI) complicated by severe left ventricular dysfunction, and hypertransaminasemia (grade 3).

During hospitalization, the patient was undergoing to coronary angiography and subsequently coronary CT scan without evidence of relevant stenosis or acute plaque rupture, thus excluding an acute coronary syndrome.

Importantly, echocardiography revealed a complete, apical and midventricular akinesia and left ventricular dysfunction (ejection fraction of 38%) associated to elevated cardiac markers such as brain natriuretic peptide and cardiac troponin I. The cranial CT scan showed no signs of ongoing bleeding.

The patient was not subjected to further investigation with cardiac magnetic resonance.

These imaging features were suggestive of Takotsubo cardiomyopathy, however, immunotherapy-related myocarditis could not be excluded.

To confirm the suspected diagnosis of Takotsubo syndrome, a later echocardiography performed at four weeks reported a recovery of the left ventricular systolic dysfunction with resolution of apical akinesia (Lyon).

Therapy for heart disease included antiplatelet drug, beta-blocker, diuretics and norepinephrine injection for acute hypotension. For the management of irAEs, immunosuppressive therapy with oral systemic steroid was initiated resulting in rapid improvement and progressive reduction of transaminases. She was discharged after fourteen days of hospitalization on prednisone 50 mg daily with plan for gradual taper over 6 weeks.

In terms of effectiveness, after only two cycles of immunotherapy, she obtained a clinical benefit with surprising complete metabolic response according to PET Response Criteria in Solid Tumors (PERCIST) (16), with disappearing of two hepatic nodules and of lung metastases to 18-FDG PET scan performed in February 2023, as shown in **Figure 1**.

**Figure 2:**
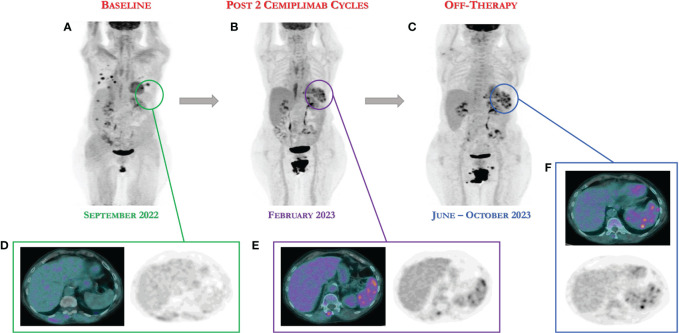
18-FDG PET/CT scan demonstrates the persistence of complete response after only two cycles of cemiplimab definitively discontinued for severe toxicities. **(A)** Baseline 18-FDG PET image. **(B)** 18-FGD PET showing complete response after two cycles of immunotherapy. **(C)** 18-FDG showing the persistence of complete response following nine and thirteen months (June and October 2023), despite the discontinuation of immunotherapy. **(D–F)** As result of an effective immune activation, the 18-FDG PET scan showed an intense increase in uptake to the spleen, a finding not present at the previous pre-immunotherapy examination.

Probably as result of an effective immune activation, the 18-FDG PET scan showed an intense increase in uptake to the spleen, a finding not present at the previous pre-immunotherapy examination (see **Figure 2**). Importantly, the spleen is the largest secondary lymphoid organ, although mechanisms underlying lymphocyte trafficking to the spleen induced by immunotherapy remain unclear.

Therefore, in consideration of the objective and metabolic response, the clinical benefit and the onset of severe and unacceptable toxicities, the immunotherapy was definitively discontinued.

Interestingly, nine and thirteen months after the start of cemiplimab treatment, the patient is off therapy and still disease-free (complete response) as documented by the last 18-FDG PET scan (see **Figure 2**). In addition, she is currently completely asymptomatic without any systemic symptoms.

## Discussion

In advanced CC setting, the strategy based on the use of platinum-based regimens is the most active in first line. Advanced CC patients who progress after fist line therapy have a poor prognosis and the subsequent available options were until recently disappointing.

In this setting, the use of ICIs has changed even the management of gynecological cancers over the last few years (17).

The immunotherapeutic approach with anti-PD-1 ICI cemiplimab led to significantly longer OS than chemotherapy among patients with recurrent CC who had had disease progression after first-line chemotherapy (18).

Here we report the first case of patient with pre-treated advanced CC with PD-L1 expression of less than 1%, effectively treated with cemiplimab as a part of a compassionate use in a managed access program.

Our decision to manage this case with cemiplimab was based on the positive results of the phase III EMPOWER-Cervical 1 trial (13).

This trial showed promising improvement in the OS of patients treated with immunotherapy compared to single-agent chemotherapy in patients regardless of their PD-L1 status.

To date the PD-L1 expression is the only immune-related biomarker investigated in CC patients that could potentially predict benefit to immunotherapy. In the pivotal trial, among the patients with PD-L1 expression of 1% or greater, median OS was 13.9 months with cemiplimab, as compared with 9.3 months with chemotherapy. Instead, among the patients with PD-L1 expression of less than 1% median OS was 7.7 months with cemiplimab and 6.7 months with chemotherapy (13).

In the overall population, an objective response occurred in 16.4% in the cemiplimab group, as compared with 6.3% in the chemotherapy group. An objective response occurred in 18% of the cemiplimab-treated patients with PD-L1 expression ≥1% and in 11% of those with PD-L1 <1% (13).

These results suggest that some patients with PD-L1 negative may still have a benefit to cemiplimab, as we demonstrate in our clinical case. The main best overall tumor response to cemiplimab was the stable disease (41.4%). In our clinical case we report an early and complete response to cemiplimab that occurred in only 3.3% in the pivotal trial.

Unfortunately, the immune system activation may lead to a novel type of toxicities known as irAEs (19). These events may potentially involve any organ system with variable clinical presentation and prognosis (20). Specifically, cardiovascular irAEs are rarely reported with an incidence of 1.3% (21) but are often severe and can be life threatening. Myocarditis represents the most frequent immune-related cardiovascular toxicity (22). Pericarditis, arrhythmias, and pericardial effusion are less frequent. Typically, the incidence is higher with the combined use of different ICIs, although it has been reported to occur with a single ICI.

Regarding the safety profile of cemiplimab, in EMPOWER-Cervical 1 the irAEs of grade 3 or higher occurred in 45% in the cemiplimab group (13). The irAEs that resulted in discontinuation of the trial treatment occurred in 8.7% receiving cemiplimab. The increase in transaminases of any grade were described in only 2.7% of cemiplimab group and 0.7% of severe grade. Interestingly, no cardiovascular irAEs were reported by the investigators (13). Therefore, this is the first clinical case in which an immune-related cardiological toxicity such as Takotsubo syndrome is described. As this is a relatively uncommon ICI-associated cardiotoxicity, to date there are not many data in the literature regarding the incidence and prognosis (23).

Although no data are available regarding the correlation between the occurrence of severe irAEs and the effectiveness of cemiplimab in the setting of advanced CC, we describe for the first time the case of a patient undergoing immunotherapy who showed an exceptional and early response concomitantly with the onset of severe and rare irAEs. Indeed, it has been hypothesized that irAEs, especially in skin, endocrine organ or gastrointestinal tract are related to a significant survival benefit (24). This correlation has been widely described in patients with melanoma and lung cancer treated with ICIs (25, 26).

Another interesting aspect of this case is represented by the persistence of clinical benefit and disease progression-free for thirteen months, despite the early immunotherapy discontinuation (27). There are several reports that anecdotally indicate the durable response for the patients who discontinued immunotherapy due to toxicity, but no data with cemiplimab in CC setting.

Therefore, in the event of immunotherapy discontinuation due to irAEs, one should not immediately switch to a subsequent line of therapy especially in the case of clinical benefit or subsequent evidence of radiological response.

Finally, in our patient perhaps as result of an excessive and effective immune activation, the 18-FDG PET scan showed an intense increase in uptake to the spleen following the onset of severe immune toxicity. Regarding this, it has been reported that the PET scan can potentially monitor the metabolic changes in peripheral lymphoid organs such as lymph nodes and spleen (28). The spleen is the largest secondary lymphoid organ, while the mechanisms underlying lymphocyte trafficking remain unclear. Thus, it has been hypothesized that PET examination can predict the efficacy of immunotherapy identifying patients with effective immune activation.

Anyway, the optimal approach to detect the metabolism of peripheral lymphoid organs through the 18-FDG PET and to predict the therapeutic effect of ICIs is yet to be established.

## Conclusion

To our knowledge, we report for the first time a case of advanced CC refractory to standard of care platinum-based with a complete and early metabolic response after only two cycles of immunotherapy with cemiplimab.

Our case provides unequivocal clinical evidence for the immunotherapy effectiveness in treating CC even in patients with no tumor PD-L1 expression.

Importantly, this case highlights that the therapeutic effect of cemiplimab could be maintained for a long period after early discontinuation of its administration, thus suggesting a potential correlation between the activity of immunotherapy and the onset of severe and rare irAEs.

## Data availability statement

The original contributions presented in the study are included in the article/supplementary material. Further inquiries can be directed to the corresponding author.

## Ethics statement

Written informed consent was obtained from the individual(s) for the publication of any potentially identifiable images or data included in this article.

## Author contributions

AP: Conceptualization, Data curation, Writing – original draft, Writing – review & editing. CP: Validation, Visualization, Writing – review & editing. EC: Validation, Visualization, Writing – review & editing. JV: Validation, Visualization, Writing – review & editing. SCC: Validation, Visualization, Writing – review & editing. MDN: Validation, Visualization, Writing – review & editing. LC: Data curation; Visualization, Writing – review & editing. SL: Data curation; Visualization, Writing – review & editing. SP: Conceptualization, Writing – original draft, Writing – review & editing.
